# Dynamics of Cholera Outbreaks in Great Lakes Region of Africa, 1978–2008

**DOI:** 10.3201/eid1711.110170

**Published:** 2011-11

**Authors:** Didier Bompangue Nkoko, Patrick Giraudoux, Pierre-Denis Plisnier, Annie Mutombo Tinda, Martine Piarroux, Bertrand Sudre, Stephanie Horion, Jean-Jacques Muyembe Tamfum, Benoît Kebela Ilunga, Renaud Piarroux

**Affiliations:** Université de Franche-Comté, Besançon, France (D. Bompangue Nkoko, P. Giraudoux, M. Piarroux, B. Sudre); Ministère de la Santé Publique, Kinshasa, Democratic Republic of Congo (D. Bompangue Nkoko, A. Mutombo Tinda, J.-J. Muyembe Tamfum, B. Kebela Ilunga); Royal Museum for Central Africa, Tervuren, Belgium (P.-D. Plisnier); Joint Research Centre of the European Commission, Ispra, Italy (S. Horion); Université de Kinshasa, Kinshasha (J.-J. Muyembe Tamfum); Université de la Méditerranée, Marseille, France (R. Piarroux); University Hospital La Timone, Marseille (R. Piarroux)

**Keywords:** cholera, epidemics, Vibrio cholerae, diarrhea, outbreak, transmission, epidemiology, bacteria, Democratic Republic of the Congo, Kenya, Uganda, Rwanda, Burundi, Tanzania, Africa, El Nino, climate, phytoplankton, plankton, research

## Abstract

Cholera outbreaks have occurred in Burundi, Rwanda, Democratic Republic of Congo, Tanzania, Uganda, and Kenya almost every year since 1977–1978, when the disease emerged in these countries. We used a multiscale, geographic information system–based approach to assess the link between cholera outbreaks, climate, and environmental variables. We performed time-series analyses and field investigations in the main affected areas. Results showed that cholera greatly increased during El Niño warm events (abnormally warm El Niños) but decreased or remained stable between these events. Most epidemics occurred in a few hotspots in lakeside areas, where the weekly incidence of cholera varied by season, rainfall, fluctuations of plankton, and fishing activities. During lull periods, persistence of cholera was explained by outbreak dynamics, which suggested a metapopulation pattern, and by endemic foci around the lakes. These links between cholera outbreaks, climate, and lake environments need additional, multidisciplinary study.

## Abstract 2

Outbreaks fluctuate on the basis of season, rainfall, plankton bloom, and fishing activities.

In Asia, the endemic and seasonal character of cholera largely depends on human exposure to the aquatic reservoirs of *Vibrio cholerae* (*1*). Culturable *V. cholerae* as well as viable but nonculturable *V. cholerae* (i.e., those that have entered into a dormant stage because of conditions unfavorable for growth or reproduction) attach to zooplankton and phytoplankton, especially in estuarine areas (*2*). In these areas, the incidence of cholera is influenced by local factors, such as rainfall and plankton blooms, and by global climatic conditions, such as increased sea surface temperatures linked to El Niño Southern Oscillation events (*3**,**4*). This link between cholera, the aquatic environment, and climate, named the “cholera paradigm” by Colwell (*5*), was highlighted by numerous studies in coastal areas.

Except for Haiti, where an epidemic of cholera began in mid-October 2010 (*6*), the area experiencing the worst cholera epidemics is sub-Saharan Africa. During 1995–2005, a total of 632 cholera outbreaks were reported worldwide; 66.0% of the total cases and 87.6% of fatal cases were reported from sub-Saharan Africa (*7*). Specifically, according to the World Health Organization (WHO), only 5 countries (Burundi, Cameroon, the Democratic Republic of the Congo [DRC], Ghana, and Tanzania) have reported cases of cholera every year since 1990 (*8*). Three of these countries—Burundi, DRC, and Tanzania—are partially or totally located in the African Great Lakes region (AGLR), an area including Lakes Tanganyika, Victoria, Kivu, Edward, and Albert. This region also includes Rwanda and part of Kenya and Uganda, which have also reported cases of cholera nearly every year since 1991 (except for 3 years for Kenya and 2 years for Rwanda and Uganda). Except for some limited epidemics, AGLR was long free from cholera, which emerged in 1977–1978 when the 6 countries were simultaneously affected (*9*). Since then, AGLR has become one of the most active foci of cholera, declaring 322,532 cases during 1999–2008 (20% of all cholera cases officially reported worldwide to WHO for these 10 years). Nevertheless, these numbers are widely underestimated because many patients cannot access health care facilities (*10*). This worrying evolution of cholera outbreaks in AGLR went unnoticed in the scientific community, and no serious attempts have been made to describe these new endemic foci of a waterborne disease originating from coastal marine environments.

Considering the established link between rainfall, El Niño events, sea surface temperature, plankton, and cholera in the coastal areas, the emergence of cholera and its spread in AGLR can be hypothesized to have been facilitated by global climatic and local environmental factors. However, the AGLR environment differs widely from estuarine environments, which are known to harbor favorable ecosystems for *V. cholerae* survival during interepidemic periods (*1**–**5*). Our study aimed to describe 1) cholera outbreak dynamics in the AGLR, 2) the modes of persistence of *V. cholerae* during lull periods, and 3) the role of specific climatic conditions that might trigger widespread epidemics.

## Methods

### Data Collection and Case Definitions

Annual reports of cholera cases during 1978–2008 in Burundi, Rwanda, DRC, Tanzania, Uganda, and Kenya were retrieved from a WHO website (*8*), but it provided information only to the country level. To obtain more detailed information, we also referred to the ProMED website (*11*), which compiles information about cholera outbreaks reported by official government and international agencies, print and online media, and local observers. However, ProMED can miss some outbreaks and possibly bias the spatial distribution toward areas with major outbreaks. In addition, outbreak data on the website are not always accurate. Therefore, with the help of local and national staff of the DRC Ministry of Health, information about cholera cases was collected weekly in each DRC health district during 2002–2008. Attack rates were calculated by using population data provided by the Congolese Ministry of Health. Cholera cases were diagnosed on the basis of the WHO standard case definition (i.e., acute watery diarrhea, with or without vomiting, in a patient >5 years of age). In DRC, the national surveillance system lowers the age limit to 2 years for case-patients associated with confirmed cholera outbreaks. Samples for only a small percentage of suspected cholera case-patients were submitted for laboratory confirmation. Nevertheless, as recommended by WHO, outbreaks are usually confirmed by culture results and by identification of *V. cholera* O1 in fecal samples. This confirmatory testing is performed by national health staff, sometimes with the support of staff from WHO or international nongovernmental organizations. For instance, in 2009 WHO helped to confirm 38 cholera outbreaks in Africa (*12*).

### Statistical Analysis and Geographic Information System

Pearson correlations were computed between the time series of annual data of cholera cases in the 6 AGLR countries (*13*). Significance was estimated by computing H_0_ (the null hypothesis) probability using the Monte Carlo method (999 replicates).

A geographic information system was established on the basis of data collected in the 515 DRC health districts during 2002–2008. Six health districts were not included in the statistical analysis because >10% of their weekly reports were missing. We examined the relationship between the number of cholera cases in each health district and the following variables: population, presence or absence of railways, presence or absence of roads, and lakeside location. Populations of each health district were log-transformed, and log(population) was included as an offset term in the model. Because of the overdispersion of cholera incidence, several generalized linear models belonging to the negative binomial family were compared and checked for spatial structure. Stepwise selection of variables was performed in each case, and the best models were selected by using the Akaike index criterion, following Venables and Ripley (*14*) and Rigby and Stasinopoulos (*15*). We checked model residuals for spatial structure by plotting an empirical variogram. A variogram envelope was then computed by performing 1,000 permutations of the residual values on the spatial locations (the health district centroids). All semivariances that were observed were within the limits of the envelope, indicating that no spatial correlation could be detected in the residuals.

To investigate for case clustering, we used SaTScan software (Kulldorf, Cambridge, UK) to analyze the case numbers in each Congolese health district during 2002–2008. To detect clusters, this software systematically moves a circular scanning window of increasing diameter over the studied region and compares observed case numbers inside the window to the numbers that would be expected under the null hypothesis (i.e., a random distribution of cases) (*16*). The radius of the maximum allowed cluster size was 200 km. The significance for each cluster was obtained through Monte Carlo hypothesis testing (i.e., results of the likelihood function were compared with 999 random replications of the dataset generated under the null hypothesis) (*17**,**18*).

Time series of cholera cases that occurred in the health district belonging to the main clusters identified by the Kulldorf method were decomposed into a trend, a seasonal component, and a residual by using a seasonal-trend decomposition procedure based on Loess regression following the method of Cleveland et al. (*19*). Cross-correlations between time series were computed, and health zones with synchronous patterns were grouped into 5 hotspots.

To investigate the possible link between cholera and rainfall, we analyzed the rainfall time series obtained for the 5 hotspots from January 1, 2002, through December 31, 2008, and decomposed the time series into trend, seasonal, and residual components as explained by Venables and Ripley (*14*). We then checked for a correlation between the residual components of rainfall and cholera data. Rainfall data were obtained from the International Research Institute for Climate and Society IRI/LDEO Climate Data Library, providing the estimated daily precipitation in Africa from the National Oceanic and Atmospheric Administration Climate Prediction Center (*20*). The daily estimated precipitation from January 1, 2002, through December 31, 2008, was extracted for 5 areas, including the 5 hotspots, and aggregated on a weekly basis. The areas were North Kivu (28.7°–29.7°E, 1.2°–1.7°S), South Kivu (28.7°–29.2°E, 1.7°–2.2°S), Uvira (28.6°–29.3°E, 2.6°–3.9°S), Kalemie (28.1°–29.5°E, 5.6°–7.2°S), and Upper Congo Basin (25.5°–26.6°E, 8.0°–9.9°S).

The link between dynamics of cholera and fluctuation of phytoplankton in Lake Tanganyika was studied by using remote sensing data of chlorophyll-*a* (in μg/L) and field measurements from 2002 through 2006 (*21**–**23*). This dataset, which was computed by using daily MODIS/Aqua Level 1B images (http://oceancolor.gsfc.nasa.gov), was specifically optimized for the monitoring of plankton blooms in Lake Tanganyika (*21*). Chlorophyll-*a* is a usual proxy for phytoplankton concentration (*24*). Whole-lake chlorophyll-*a* data were specifically investigated near Uvira (3°23′18′′ S, 29°12′27′′ E) and Kalemie (5°55′91′′ S, 29°15′00′′ E) for this study. Computations were done and graphical displays were made by using R 2.12.2 (*25*), with MASS 7.3–11 and GAMLSS 4.0–8 (both from The R Foundation for Statistical Computing, Vienna, Austria), and by using ArcGIS 9.3 (Environmental Systems Research Institute, Inc., Redlands, CA, USA). Finally, because human activities may also influence the seasonal pattern of cholera, we conducted field observations and systematic interviews in each hotspot to understand the lifestyles of fishermen, tradesmen, artisans, and other inhabitants of the region.

## Results

### Temporal Dynamics of Cholera and El Niño Warm Events

**Figure 1:**
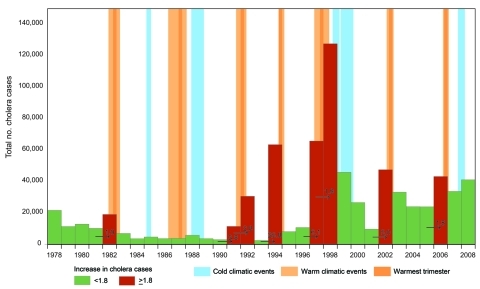
Yearly number of cholera cases in the African Great Lakes region (Burundi, Democratic Republic of Congo, Kenya, Rwanda, Tanzania, and Uganda), 1978–2008. Red bars indicate years with large increases in cholera cases. Numbers on arrows represent the increase factor in cholera cases. Warm climatic events (indicated by light orange background) had a duration of >5 months and a sea surface temperature increase of >0.5°C simultaneously in Niño 3 (eastern Pacific, from 90°W–150°W and 5°S–5°N) and Niño 4 (western Pacific, from 160°E–150°W and 5°S–5°N) regions.

The annual cholera cases for 1978–2008 for Burundi, DRC, Tanzania, Uganda, and Kenya (but not for Rwanda) were synchronized without a time lag (Table). We found a large increase in cholera for 8 years (the numbers in parentheses after the years show the increase over the preceding year): 1982 (1.9×), 1991 (3.8×), 1992 (2.8×), 1994 (25.8×), 1997 (6.1×), 1998 (1.9×), 2002 (5.0×), and 2006 (1.8×) (Figure 1). By extracting El Niño southern oscillation events indices from the National Oceanic and Atmospheric Administration website (www.cpc.ncep.noaa.gov/data/indices/wksst.for), we found 7 warm events (abnormally warm El Niños). These events lasted >5 months and corresponded to periods during which the monthly sea surface temperature exceeded the expected sea surface temperature by at least 0.5°C at the same time in the Niño 3 and Niño 4 regions. The 7 warm events peaked during the last trimester of 1982; the third trimester of 1987; the first trimester of 1992; and the last trimesters of 1994, 1997, 2002, and 2006, which exceeded the expected sea surface temperature by 1.81°C, 1.28°C, 1.14°C, 1.01°C, 2.27°C, 1.26°C, and 1.16°C, respectively. All but 1 of these warm events corresponded to the years cited above that had large increases in cholera; thus, we tested the hypothesis that this was a random occurrence but found that to be an unlikely hypothesis (p = 0.0003, Fisher exact test).

**Table:** Annual correlations for cholera cases between 6 countries in the African Great Lakes region, 1978–2008*

Country	Correlation coefficient (p value)					
	Burundi	DRC	Kenya	Rwanda	Tanzania	Uganda
Burundi						
DRC	**0.4937 (0.0048)**					
Kenya	**0.4789 (0.0064)**	0.3133 (0.0861)				
Rwanda	0.1307 (0.4833)	0.2665 (0.1473)	0.168 (0.3665)			
Tanzania	0.327 (0.0725)	0.1721 (0.3545)	**0.4338 (0.0148)**	0.2792 (0.1282)		
Uganda	**0.5631 (0.001)**	**0.7284 (0.00001)**	**0.4304 (0.0157)**	0.2884 (0.1157)	**0.5076 (0.0036)**	

*Values in **boldface** are significant. DRC, Democratic Republic of Congo.

### Cholera Epidemics and Lakeside Area

**Figure 2:**
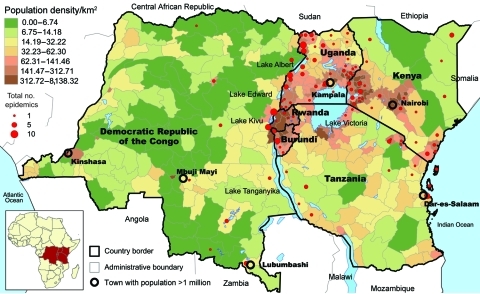
Number of reports and localization of cholera outbreaks in the African Great Lakes region, 1999–2008, as reported by ProMED (*11*).

**Figure 3:**
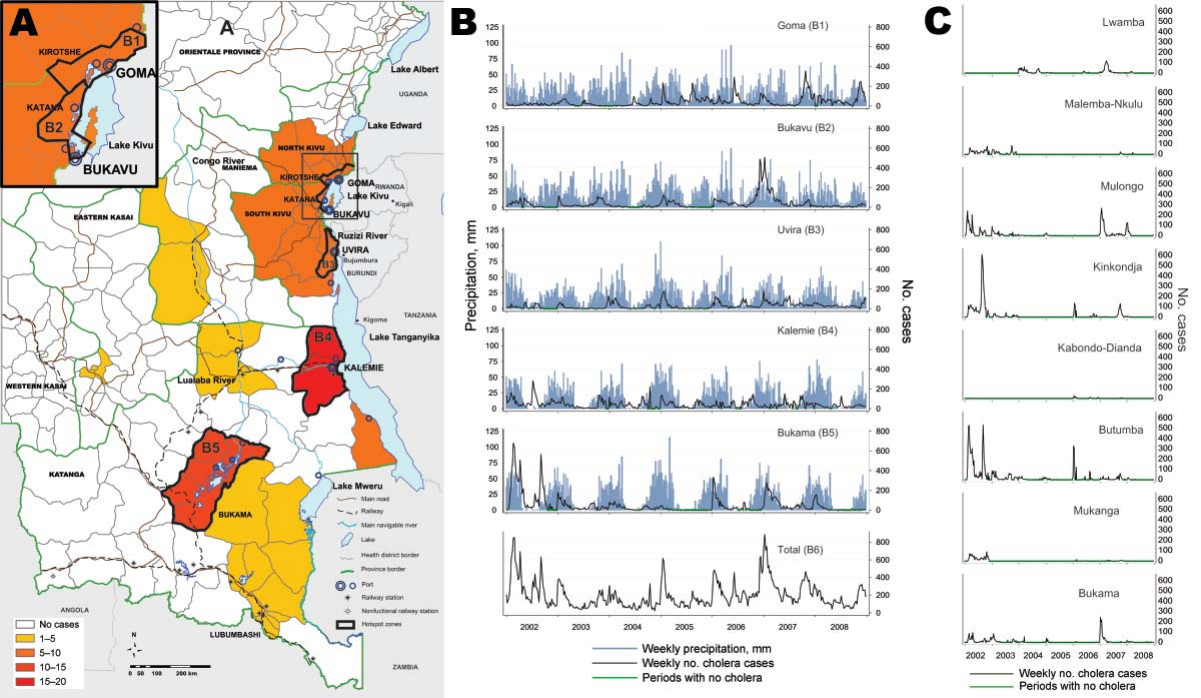
Temporal-spatial evolution of cholera cases in 5 hotspots in the African Great Lakes region, 2002–2008. A) Spatial distribution of cholera in the provinces of Katanga, North Kivu, and South Kivu (Democratic Republic of Congo). Health districts are colored according to the risk ratio of the cluster, as calculated by using SatSCan software (Kulldorf, Cambridge, UK). B) Evolution of the weekly number of cholera cases in the 5 hotspots (B1–B5). B1) Goma and Kirotshe health districts; B2) Bukavu and Katana health districts; B3) Uvira health district; B4) Kalemie and Nyemba health districts; B5) 8 health districts in the Upper Congo River Basin (see district names in panel C); B6) total cases for the 5 hotspots. Green indicates periods without cholera; blue indicates estimated weekly rainfalls. The global curve did not show any remission periods. C) Evolution of the weekly number of cholera cases in the 8 health districts composing the Upper Congo Basin hotspot. The epidemic curve in B5 was composed of partially synchronous epidemics separated by periods of lull.

Using the ProMED website, we identified and localized 252 cholera epidemics for 1999–2008 (Figure 2) (*11*). Of the outbreaks, 63.5% occurred in districts in lake areas, mainly around Lakes Victoria, Kivu, Albert, and Edward and the northern half of Lake Tanganyika. By contrast, only 12% of outbreaks occurred in seaside areas of Kenya and Tanzania. We then analyzed data provided by DRC, which reported 159,086 cholera cases and 4,912 cholera-related deaths during 2002–2008, corresponding to 66% of the cases and 71% of the deaths reported to WHO from the 6 AGLR countries. Using the type II negative binomial model (lowest Akaike information criterion and sigma coefficient = 8) and including the presence of roads and lakeside location, we found that the number of cholera cases in each health district in DRC was significantly higher in areas with roads (risk ratio [RR] 1.4, 95% confidence interval 1.1–1.9) and lakes (RR 7.0, 95% confidence interval 4.9–10.0). Results of the SaTScan analysis showed that the spatial clusters that were associated with significant RRs were all located in eastern DRC (Figure 3). The 3 clusters with maximal RR (p<0.001) were 1) Kalemie, on the shore of the Lake Tanganyika (RR 17.1); 2) the area bordering lakes on the Upper Congo basin (RR 12.7); and 3) an area including the northern shore of Lake Tanganyika, Lake Kivu, and the southern shore of Lake Edward (RR 6.0). These 3 clusters represented 28 health districts and 107,826 cases of cholera (68% of the total cases and <10% of the total population of DRC). When considering these 3 clusters altogether, cholera cases were reported every week during the 7-year period studied. The lowest incidence was 8 cases per million inhabitants (week 24, 2004). By contrast, outside of these 3 clusters, we identified numerous periods with no or almost no cholera cases (<1 case/1 million inhabitants/week), many of them lasting >1 month (weeks 24–30, 2002; weeks 19–28 and 36–40, 2004; weeks 9–13, 20–34, and 44–49, 2005; weeks 16–25, 2006; and weeks 23–27, 2007).

By using time-series analysis to search for synchronous patterns, we identified 5 hotspots within these spatial clusters, accounting for 84,465 cholera cases. The first hotspot was around Goma in North Kivu (including Goma and Kirotshe health districts; Figure 3); the second hotspot was around Bukavu in the north of South Kivu (Bukavu and Katana health districts); the third hotspot was in Uvira, in the south of South Kivu (Uvira health district); the fourth hotspot was around Kalemie, near Lake Tanganyika in Katanga (Kalemie and Nyemba health districts); and the fifth hotspot was in the Upper Congo River Basin in Katanga (Bukama, Butumba, Kinkondja, Kabondo-Dianda, Malemba-Nkulu, Lwamba, Mukanga and Mulongo health districts). In each of these hotspots, cholera cases were reported almost every week except for a few short interruptions (Figure 3).

### Cholera Weekly Incidence by Season, Rainfall, Plankton Abundance, and Fishing Activities

**Figure 4:**
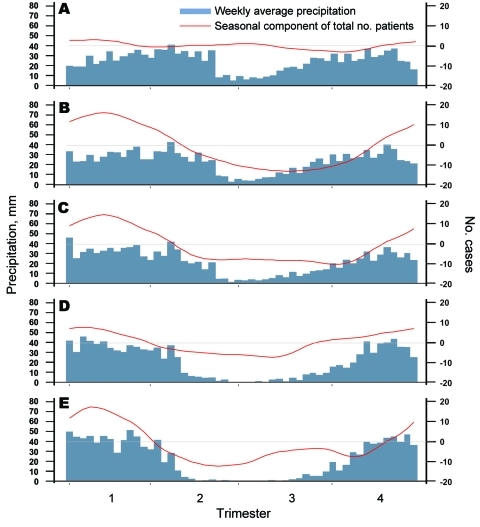
Seasonal patterns/components of cholera outbreaks for 5 hotspots in the African Great Lakes region, 2002–2008. Hotspots are Goma (A), Bukavu (B), Uvira (C), Kalemie (D), and Upper Congo Basin (E). Blue indicates the weekly average precipitation (in mm); red indicates the seasonal component of the total number of patients after the time series was decomposed into a trend and seasonal and residual components by using a seasonal-trend decomposition procedure based on loess regression. Horizontal gray lines indicate seasonal component = 0.

Seasonal patterns of cholera varied according to the location of the hotspots (Figure 4). Around Goma, where no dry season could be determined, time-series analyses did not identify any seasonal component in the occurrence of cholera. Around Bukavu and Uvira—2 hotspots characterized by a short dry season—a clear trend toward a lull in cholera cases during the dry season was observed. Further south, in Kalemie and the Upper Congo River Basin, cholera outbreaks started before the end of the dry season and worsened during the rainy season.

Cross-correlations between residual components of cholera and rainfall time series showed a significant positive relationship in Uvira after a latency of 2–5 weeks and in the Upper Congo River Basin after no latency. In Kalemie and Bukavu, the link between rainfall and cholera was supported only by the seasonal trend. Therefore, the deleterious effect highlighted during the El Niño years might, at least partly, have resulted from excess rainfall in the Great Lakes region.

**Figure 5:**
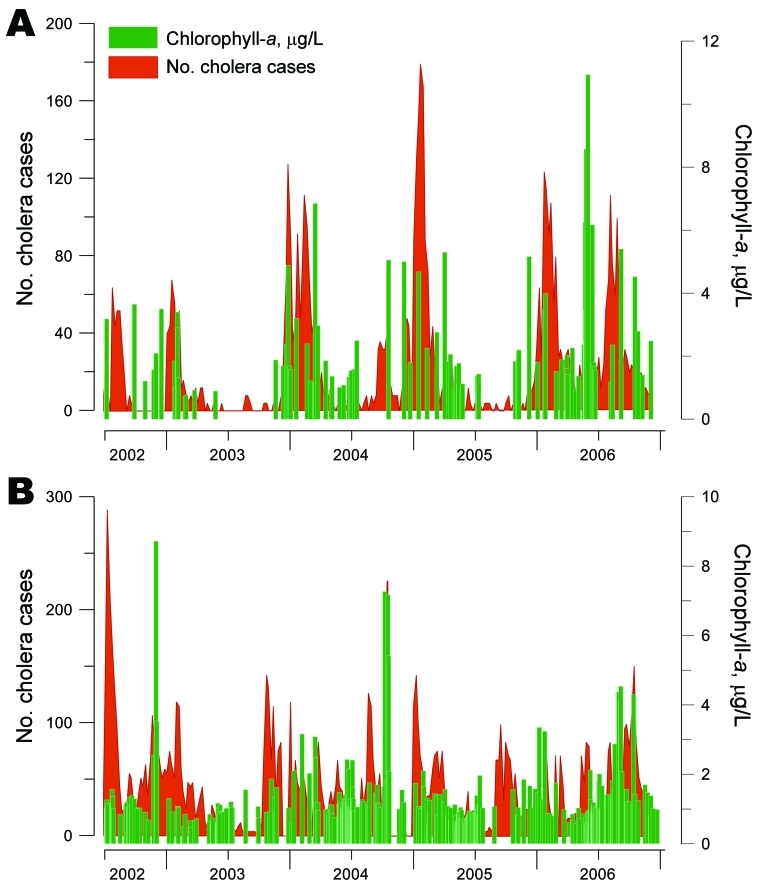
Link between the number of cholera cases and fluctuations in phytoplankton abundance (chlorophyll-*a* concentrations) in Lake Tanganyika, Africa Great Lakes region, January 2002–December 2006. Two of 5 cholera hotspots in the region were tested, both of which face Lake Tanganyika: Uvira (A) and Kalemie (B). Green indicates median concentrations of chlorophyll-*a* in surface water; red indicates cholera cases.

We further studied the links between the dynamics of cholera and the fluctuations of phytoplankton in Lake Tanganyika by using chlorophyll-*a* concentration estimates (in μg/L) derived from remote sensing. Cholera epidemics and blooms of phytoplankton occurred almost simultaneously in Uvira and Kalemie (Figure 5). However, after removal of the seasonal components of the time series, no additional significant relationship was found between these 2 phenomena.

**Figure 6:**
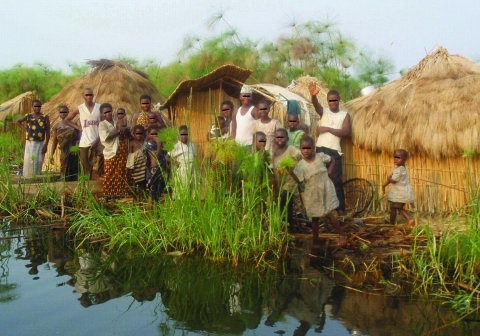
Fishing camp on an island in Lake Upemba, upper Congo River Basin. Fishermen and their families usually spend several weeks every year in camps like this, in which the lake is the only source of water. Because there is no firewood in such areas, campaigns promoting the boiling of water are useless. (Photograph by Didier Bompangue.)

In Kalemie and the Upper Congo River Basin, which are among the main fishing areas in DRC, field investigations and interviews focused on descriptions of the behaviors of fishermen and of the seasonal variations in trading and fishing activities. In Kalemie, fishing activities peaked from mid-July to September (the dry season), when fishermen move into settlements located on the shore of Lake Tanganyika. In the Upper Congo River Basin, the fishing season is slightly earlier (mid-June to September), and fishermen crowd into camps on islands that emerge during the dry season but are below water during the rainy season. These fishing settlements are characterized by poor sanitary conditions, which lack clean water and a system for disposing of excreta (Figure 6). In both areas, the mild increase in cholera cases during the dry season is associated with the traveling of fishermen and traders between the main towns and fishing camps.

### Persistence of Cholera during Lull Periods

Because of the combined effects of seasonal patterns and interannual trends of cholera, short lulls in cholera outbreaks occurred in the 5 hotspots. However, these lull periods were not completely synchronous (Figure 3). Although the number of cholera cases fell to zero in a given hotspot, neighboring hotspots were still undergoing outbreaks and served as starting points for cholera to recolonize other lakeside areas. The high and still increasing density of population has resulted in less frequent and shortened periods with complete interruption of cholera transmission in Kalemie, Uvira, Bukavu, and Goma, adding to the stability of this pattern of epidemics (Figure 3). Therefore, spontaneous and simultaneous extinction in every hotspot was never observed during this 7-year survey.

## Discussion

Our findings show that cholera in AGLR greatly increases during years of El Niño warm events, and it decreases or remains stable between these warm events. In this region of Africa, which is located near the equator, rainfall affects the epidemiologic patterns of cholera. Therefore, the deleterious effect during El Niño warm event years might at least partly result from the excess of rainfall during the corresponding years. Seasonal patterns of cholera and the effects of the rainfall shown by our results corroborate the findings from a study performed in Zambia, a country bordering the southern Katanga Province, DRC (*26*). There, the risk for cholera epidemics increases when the rainy season begins earlier and is preceded 6 weeks earlier by a period of warm temperature.

Our results also show that a few lakeside areas play a crucial role in maintaining endemic cholera in AGLR. Two case-control studies, including 1 on Lake Tanganyika (*27*), showed a statistical correlation between contracting cholera and living on the shores of a lake or a river in Africa (*27**,**28*). The link between high incidence of cholera and presence of lakes has also been noted in DRC at the provincial level (*29**,**30*). We addressed this issue through a multiscale approach and obtained data suggesting that lakeside areas were the source of the disease in the entire AGLR. Indeed, lakeside areas were the only areas where the disease persisted continuously during the study period. Therefore, we believe that in the absence of lakeside areas, the disease would have disappeared from AGLR.

Two hypotheses emerged to explain how cholera took root in AGLR, an area far from the coastal marine environments known to be the original biotope of *V. cholerae*. The first hypothesis involves the possible persistence of some cholera strains in the lakes of AGLR. Weather conditions (i.e., seasonal rainfall and the multiannual recurrence of El Niño warm events) might promote plankton growth and *V. cholerae* multiplication, similar to the epidemiology of cholera in South Asia (*3*). Climatic changes have resulted in biological modifications of the lakes. The temperature of the African Great Lakes has increased during the past 3 decades (*31**–**33*). Changes in algal community structure have also occurred; for example, the reported Lake Tanganyika cyanobacteria-chrysophytes-chlorophytes community of 1975 was replaced by a cyanobacteria-chlorophytes-diatom community (*34*). These environmental changes, which were observed in Lakes Victoria, Malawi (another African Great Lake, also known as Lake Nyasa), and Tanganyika, could have affected the dynamics of cholera. Although our results showed a relationship between the abundance of phytoplankton and the number of cholera cases, we acknowledge that we did not demonstrate a causal relationship. Others causes, such as seasonal rainfall, may explain increased plankton bloom (because of an increase in nutrients) and increased cases of cholera (due to fecal contamination of lake water). Seasonal patterns of cholera around the lakes may also be partly explained by the seasonal variation of human exposure to aquatic reservoirs of *V. cholerae*, especially in fishing settlements.

The second hypothesis explains the persistence of cholera during the lull periods by outbreak dynamics evoking a metapopulation pattern (cholera stability on a regional scale originates from interactions between asynchronous local foci prone to extinction) and by densely populated endemic foci around the lakes. Most of these foci are towns in which humans live in close proximity to each other with poor hygiene conditions and little access to clean water. In such situations, cholera could persist during the dry season through a mix of human-to-human and waterborne transmission. Even if the African Great Lakes lack cholera strains that persist for extended periods, sewage seeping into the lakes from the towns and camps may result in transient but repetitive contamination of the water, which many AGLR residents use for cooking and drinking. In addition, cholera epidemics among fishing communities help maintain a human reservoir of the disease. At the end of the lull periods, the spread of cholera is then favored by several factors, including rainfall, which enhances water contamination, and commercial activities, which facilitate the spread of the disease.

To further understand the mechanisms and conditions that enabled cholera to take root in AGLR, an interdisciplinary study will investigate the role of freshwater environments and climatic factors in cholera dynamics in this region of Africa. This study, named CHOLTIC, is just beginning around Lake Tanganyika and involves specialists in various topics, including epidemiology, microbiology, limnology, hydrodynamics, phytoplankton, zooplankton, fisheries, remote sensing, and modeling. Our initial results support a link between cholera outbreaks, climate, and lake environment, and provide an encouraging basis for further investigation.
